# Transcutaneous Afferent Patterned Stimulation Therapy Reduces Hand Tremor for One Hour in Essential Tremor Patients

**DOI:** 10.3389/fnins.2020.530300

**Published:** 2020-11-12

**Authors:** Jai Y. Yu, Apoorva Rajagopal, Judy Syrkin-Nikolau, Sooyoon Shin, Kathryn H. Rosenbluth, Dhira Khosla, Erika K. Ross, Scott L. Delp

**Affiliations:** ^1^Cala Health, Inc., Burlingame, CA, United States; ^2^Personal Care Neurology, Oakland, CA, United States; ^3^Department of Bioengineering, Stanford University, Stanford, CA, United States

**Keywords:** neuromodulation, essential tremor, peripheral nerve stimulation, non-invasive stimulation, accelerometer

## Abstract

Essential tremor (ET) patients often experience hand tremor that impairs daily activities. Non-invasive electrical stimulation of median and radial nerves in the wrist using a recently developed therapy called transcutaneous afferent patterned stimulation (TAPS) has been shown to provide symptomatic tremor relief in ET patients and improve patients’ ability to perform functional tasks, but the duration of tremor reduction is unknown. In this single-arm, open-label study, fifteen ET patients performed four hand tremor-specific tasks (postural hold, spiral drawing, finger-to-nose reach, and pouring) from the Fahn-Tolosa-Marin Clinical Rating Scale (FTM-CRS) prior to, during, and 0, 30, and 60 min following TAPS. At each time point, tremor severity was visually rated according to the FTM-CRS and simultaneously measured by wrist-worn accelerometers. The duration of tremor reduction was assessed using (1) improvement in the mean FTM-CRS score across all four tasks relative to baseline, and (2) reduction in accelerometer-measured tremor power relative to baseline for each task. Patients were labeled as having at least 60 min of therapeutic benefit from TAPS with respect to each specified metric if all three (i.e., 0, 30, and 60 min) post-therapy measurements were better than that metric’s baseline value. The mean FTM-CRS scores improved for at least 60 min beyond the end of TAPS for 80% (12 of 15, *p* = 4.6e–9) of patients. Similarly, for each assessed task, tremor power improved for at least 60 min beyond the end of TAPS for over 70% of patients. The postural hold task had the largest reduction in tremor power (median 5.9-fold peak reduction in tremor power) and had at least 60 min of improvement relative to baseline beyond the end of TAPS therapy for 73% (11 of 15, *p* = 9.8e–8) of patients. Clinical ratings of tremor severity were correlated to simultaneously recorded accelerometer-measured tremor power (*r* = 0.33–0.76 across the four tasks), suggesting tremor power is a valid, objective tremor assessment metric that can be used to track tremor symptoms outside the clinic. These results suggest TAPS can provide reductions in upper limb tremor symptoms for at least 1 h post-therapy in some patients, which may improve patients’ ability to perform tasks of daily living.

## Introduction

Essential tremor (ET) is one of the most common movement disorders in adults, affecting approximately 1% of adults worldwide (Louis and Ferreira, 2010; Haubenberger and Hallett, 2018). ET often involves tremor of the upper limbs, which impairs patients’ abilities to perform activities of daily living (Poston et al., 2009; Bhatia et al., 2018). Though the etiology of ET is not fully understood, it is thought to involve pathological oscillations in the central tremor network via the ventral intermediate nucleus (VIM) of the thalamus (Raethjen and Deuschl, 2012; Haubenberger and Hallett, 2018).

Medication is the primary treatment option for ET (Deuschl et al., 2011; Ferreira et al., 2019). With consistent prescribed use, medications can provide hours of symptomatic tremor relief (Gironell et al., 1999), but provide benefit for only about 50% of patients (Sasso et al., 1991; Deuschl et al., 2011; Hedera et al., 2013). Secondary treatments include neurosurgical or focused ultrasound procedures that target the VIM, with the goal of interrupting the pathological oscillations in the central tremor network (Nazzaro et al., 2013; Elias et al., 2016). Deep brain stimulation (DBS) and lesioning of the VIM are effective, resulting in an approximately 80% reduction in tremor severity for an estimated 80% of patients (Deuschl et al., 2011; Munhoz et al., 2019). DBS, however, carries the significant risks associated with invasive surgical intervention, and both DBS and focused ultrasound procedures carry greater risks and expenses compared to pharmacological intervention (Deuschl et al., 2011; Munhoz et al., 2019; Sinai et al., 2019). ET patients for whom medication is ineffective and who do not qualify for or are not interested in focused ultrasound or surgical interventions are left with limited options for tremor control (Deuschl et al., 2011).

Recent neurophysiology research has suggested non-invasive electrical stimulation of the median nerve can evoke activity within the VIM and other regions of the central tremor network (Hanajima et al., 2004; Klostermann et al., 2009), and has led to the development of transcutaneous afferent patterned stimulation (TAPS) therapy (Lin et al., 2018; Pahwa et al., 2019) to treat tremor. TAPS consists of bursts of non-invasive electrical stimulation applied to the median and radial nerves that alternate at the frequency of a patient’s tremor. Two randomized, sham-controlled clinical trials (Lin et al., 2018; Pahwa et al., 2019) and one 3-month at-home clinical trial (Isaacson et al., 2020) have shown that 40 min of TAPS therapy reduces tremor severity and improves patients’ abilities to complete daily living tasks. However, these trials assessed tremor immediately after the end of stimulation, consistent with the clinical observation that traditional DBS quickly loses efficacy once stimulation is turned off (Lopiano et al., 2003; Temperli et al., 2003; Perera et al., 2015). It remains unknown for how long the benefits of TAPS therapy persist after the end of stimulation.

Deep brain stimulation research has shown that applying a bursting stimulation pattern alternating between two pairs of implanted electrodes may induce synaptic plasticity changes that persist after the stimulator is turned off, and can therefore provide effective tremor reduction beyond the end of stimulation (Popovych and Tass, 2012; Tass et al., 2012). Studies in other indications (tinnitus) have suggested that non-invasive, sensory bursting stimulation may have similar mechanistic carry-over effects (Popovych and Tass, 2014). These findings suggest that non-invasive, sensory bursting stimulation such as TAPS therapy may likewise offer extended tremor relief beyond the time of active stimulation.

The goal of this study was to characterize the time-profile of therapeutic benefit from TAPS therapy for up to 60 min following a stimulation session. The study was run as an open-label, single-arm study. Therapeutic benefit was quantified using both visual clinical ratings and sensor-based kinematic measures of tremor power.

## Methods

Fifteen patients with ET who met the inclusion criteria were enrolled at a single site under an Institutional Review Board-approved, non-significant risk protocol. Written informed consent was provided by each patient, and authorization for use of protected health information was signed. Patients were eligible if they were at least 22 years of age, had ET diagnosed by an internist or neurologist, and were willing to comply with all study protocol requirements as described below. Patients were excluded if they had (1) an implanted electrical medical device, such as a pacemaker, defibrillator, or deep brain stimulator, (2) mild tremor symptoms, defined as having baseline Fahn-Tolosa-Marin Clinical Rating Scale (Fahn et al., 1988) tremor scores in all rated sub-tasks less than 2 (see Efficacy Analysis below for rated tasks), (3) peripheral neuropathy affecting the tested upper extremity, (4) alcoholism, (5) existing clinical diagnoses of other known causes of tremor, including Parkinson’s disease, enhanced physiological tremor, and dystonia (verified with a focused neurological examination by the study’s onsite clinical investigator), (6) a history of epilepsy and epileptic-like conditions, (7) a history of heart rhythm problems, (8) changes in medication for tremor within one month prior to testing, (9) consumed more than 14 g of alcohol (e.g., 5 oz. wine) or 95 mg caffeine (e.g., 1 cup coffee) within 12 h of the study visit, (10) participated in another interventional clinical trial in the last 30 days which could confound the results of this study, unless approved by study supervisor, or were (11) pregnant, or (12) unable to communicate with the study staff. Patients’ medication status was documented at study enrollment. Patient demographics, ET history, and baseline tremor characteristics are summarized in Table 1.

**TABLE 1:** Patient information.

Demographics	
Age (years)	72.2 (8.6)
Sex, male	9 (60)
Race	
White	12 (80)
Asian	2 (13)
Black or African American	0 (0)
More than one race	1 (7)
Tremor history	
Age of onset (years)	41.7 (20.9)
Age of diagnosis (years)	55.0 (12.8)
Family history of ET	10 (67)
Current tremor therapy	
None	10 (67)
One medication	4 (27)
More than one medication	1 (7)
Prior tremor therapy	
Medication	10 (67)
Botulinum toxin	0 (0)
Other	0 (0)
**Clinical baseline severity ratings**	
FTM-CRS task score	
Action	2.2 (0.7)
Drawing	2.2 (1.3)
Postural hold	1.8 (0.7)
Pouring	1.6 (0.8)

*Categorical data reported as number (%). Continuous or ordinal data reported as mean (standard deviation).*

### Stimulation

**FIGURE 1:**
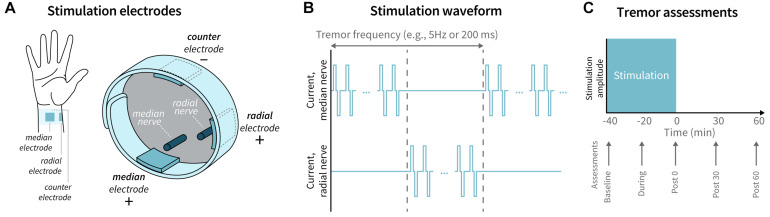
Stimulation delivery and tremor assessment timeline. **(A)** Electrodes were placed on the patient’s wrist to target the median and radial nerves, with a counter-electrode positioned on dorsal surface of the wrist. **(B)** The stimulation waveform consisted of a series of charge-balanced biphasic pulses delivered at a 150 Hz frequency, 300 μs pulse width, and 50 μs inter-pulse period, alternating between the median and radial nerve at the patient’s tremor frequency. **(C)** Tremor was assessed by visual rating and accelerometer measurements at baseline, during, immediately following (Post 0), 30 min following (Post 30), and 60 min (Post 60) following stimulation.

Patient-specific stimulation was applied unilaterally to the dominant upper limb, where dominant limb was determined by the patient’s handedness. Three hydrogel electrodes were positioned on the patient’s wrist to target the median and superficial branch of the radial nerves (Figure 1A). Active leads were placed over the median and radial nerves on the palmar surface of the wrist and were connected to a bench-top stimulator (Digitimer DS5, Digitimer, Hertfordshire, United Kingdom). A counter-electrode was connected to the dorsum of the wrist. Tremor frequency was measured using a wrist-worn accelerometer (Cala Health, Inc., Burlingame, CA) while the patient performed a 10-second forward postural hold. Stimulation consisted of a series of charge-balanced biphasic pulses, 300 μs per phase, with a 50 μs period between the two phases, delivered at a frequency of 150 Hz. The stimulation alternated between the median and radial nerve at a frequency equal to each patient’s tremor frequency (Figure 1B). This pattern was not timed to the phase of the patient’s tremor motion. The stimulation amplitude was increased by 0.25 mA increments until the patient reported paresthesia corresponding to the distributions of the median and radial nerves. The final stimulation amplitude was chosen to be the highest level of stimulation that did not elicit muscle contraction and the patient found comfortable. A stimulation session consisted of 40 min of continuous stimulation at the chosen amplitude. This stimulation protocol was consistent with protocols used in previous studies evaluating acute efficacy of TAPS (Lin et al., 2018; Pahwa et al., 2019; Isaacson et al., 2020). Safety was assessed by analyzing the reported adverse events.

### Efficacy Analysis

Tremor was evaluated at five time points: baseline (pre-stimulation), during stimulation, immediately following stimulation, 30 min after stimulation, and 60 min after stimulation (Figure 1C). At each time point, patients performed four hand-tremor specific tasks from the Fahn-Tolosa-Marin Clinical Rating Scale (FTM-CRS): postural hold, spiral drawing, finger-to-nose reach, and pouring (Fahn et al., 1988). Tremor severity was visually rated for all patients by a single expert rater according to the FTM-CRS and simultaneously measured by a tri-axial accelerometer (APDM Wearable Technologies, Portland, OR) attached to the back of the hand.

Accelerometer-measured tremor severity was quantified from the tremor power in the 3 Hz frequency band centered about the strongest tremor oscillation frequency for each task at baseline. The duration of the accelerometer signal varied between patients and tasks based on how long it took the patient to complete the task. In all cases, the first and last 10% of the accelerometer data were discarded to avoid movement artifact from transitions into and out of the tasks. The algorithm to compute tremor power included the following steps for each assessed task: (1) computing the power spectral density (PSD) of each axis of the accelerometer data using Welch’s method with a 2 second window and 50% overlap (scipy.org, signal.welch), (2) identifying the frequency of the baseline measurement’s peak tremor power in the 4–12 Hz band typically associated with ET (Haubenberger and Hallett, 2018), (3) computing the mean value of the PSD in the ±1.5 Hz frequency window centered on the frequency identified in step 2, and (4) averaging over the three axes. For each task, the association between the log_10_-transformed tremor powers and the simultaneous clinical tremor severity ratings was quantified using Spearman’s correlation coefficient.

Improvements in tremor power were expressed as “fold-improvement” ratios relative to the tremor power measured in the baseline recording for that patient and task. Thus, a reported fold-improvement of 1 indicates that tremor power is equal to the baseline power; >1 indicates an improvement (i.e., reduction) in tremor power relative to baseline; and <1 indicates a worsening (i.e., increase) in tremor power relative to baseline. Fold-improvement was chosen as the primary tremor power outcome metric to facilitate comparisons between patients and between tasks, as tremor powers vary on an exponential scale across the ET population (Elble et al., 2006). Percent improvement in tremor power relative to baseline tremor power was computed from fold-improvement as follows:

$$\begin{array}{c} \%\text{ Improvement}\equiv \frac{\text{Tremor}\;{\text{power}}_{\text{baseline}}\text{-Tremor}\;\text{power}}{\text{Tremor}\;{\text{power}}_{\text{baseline}}}\cdot 100\\ =\left(1-\frac{1}{\text{Fold-Improvement}}\right)\cdot 100 \end{array}$$

Efficacy at each time point was quantified as improvement in the average FTM-CRS score across the four tasks, and as the improvement in tremor power relative to baseline for each task. A patient was labeled as having at least 60 min of benefit beyond the end of TAPS therapy with respect to each of the five outcome metrics if that metric was better than baseline at all three post-stimulation (0, 30, and 60 min) measurements (e.g., a patient was labeled as having at least 60 min of benefit with respect to the postural hold tremor power if all three post-stimulation postural hold tremor power measurements were less than the baseline postural hold tremor power). For each of the five outcome metrics, the proportion of patients with at least 60 min of benefit was computed and tested for statistical significance using the binomial test, with a null hypothesis that 1/8 of patients would have at least 60 min of benefit (computed from the null assumption that each assessed post-stimulation time point—0, 30, and 60 min—had a ½ probability of randomly being better than baseline).

During stimulation and at each post-stimulation timepoint, patients were asked to rate the improvement of their tremor symptoms relative to their pre-stimulation severity using the Patient Global Impression of Improvement (PGI-I) scale (Busner and Targum, 2007).

## Results

**FIGURE 2:**
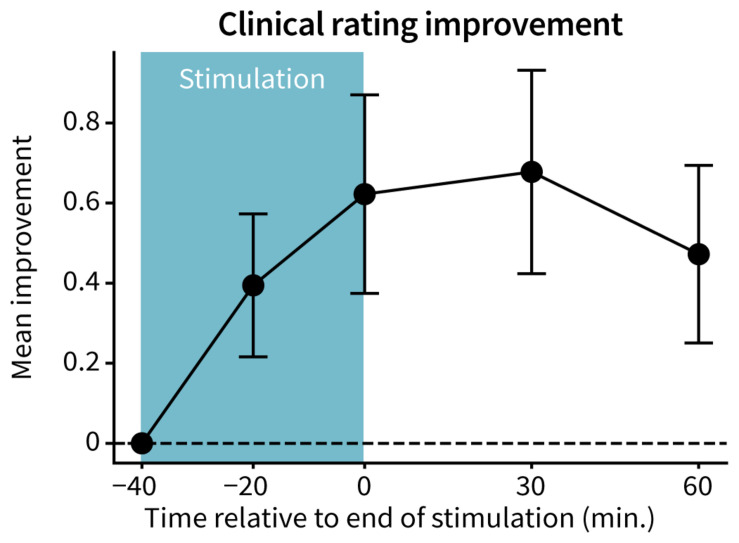
Improvement in Fahn-Tolosa-Marin Clinical Rating Scale. FTM-CRS ratings were averaged across the assessed tasks (postural hold, spiral drawing, finger-to-nose reach, and pouring) at each time point. An improvement in score indicates a reduction in tremor severity. Points and error bars represent mean ± 2 standard errors across patients (*n* = 15).

The mean FTM-CRS rating improved with TAPS therapy, with peak reductions (0.70 ± 0.25 points; mean ± 2 standard error) occurring 30 min following end of stimulation (Figure 2). 12 of 15 patients had at least 60 min of improvement in the mean clinical rating relative to baseline following end of stimulation (*p* = 4.6e-9).

**FIGURE 3:**
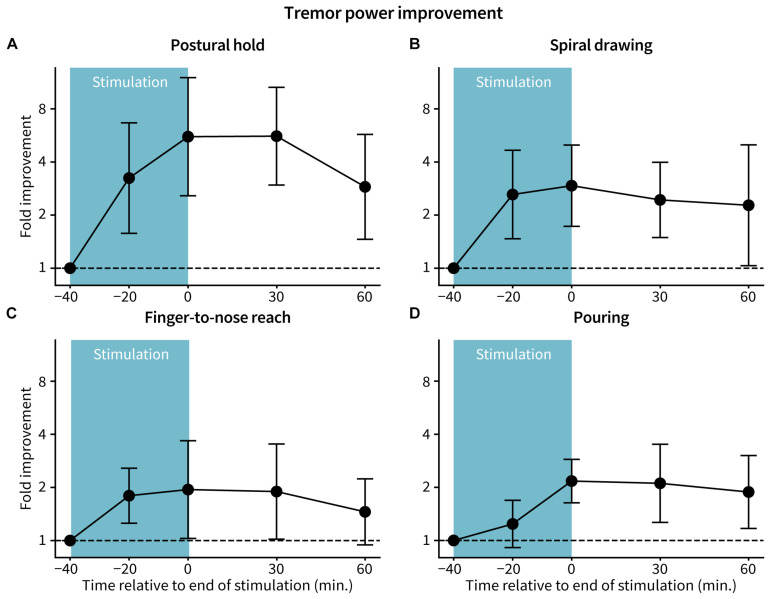
Fold-improvement in tremor power. Tremor power was calculated from accelerometer data at each assessed time for the **(A)** postural hold, **(B)** spiral drawing, **(C)** finger-to-nose reach, and **(D)** pouring tasks. Fold improvement in tremor power is the ratio between tremor power at baseline and the specified time, with a ratio >1, =1, and <1 indicating improvement, no change, and worsening from baseline, respectively. Points and error bars represent log-transformed mean ± 2 standard errors across patients (*n* = 15 for postural hold, spiral drawing, and finger-to-nose reach; *n* = 14 for pouring).

Accelerometer-measured tremor power improved with TAPS therapy across all tasks. The postural hold task had the greatest reduction in tremor power, with a median peak 5.9-fold improvement (83% reduction) in tremor power occurring 30 min following end of stimulation (Figure 3A). Median peak fold-improvements for the other tasks were 2.4 (57% reduction, spiral drawing), 1.6 (38% reduction, finger-to-nose reach), and 2.5 (59% reduction, pouring) (Figures 3B–D). 11 of 15 patients had at least 60 min of benefit following end of stimulation for each of the postural hold, spiral drawing, and finger-to-nose reach tasks (*p* = 9.8e–8) and 10 of 14 patients for the pouring task (*p* = 5.8e–7). One patient was unable to perform the pouring task due to the severity of their tremor. Individual patient tremor power trajectories for each task are shown in Supplementary Figure S1.

During stimulation, 12 of 15 patients self-reported improvements in tremor symptoms with therapy (PGI-I), with 1 patient reporting tremor “Very much improved,” 4 patients reporting tremor “Much improved,” 7 patients reporting tremor “Minimally improved,” and the remaining 3 patients reporting “No change.” Sixty minutes after the end of stimulation, 11 of 15 patients self-reported improvements in tremor symptoms (PGI-I), with 3 patients reporting “Much improved,” 8 patients reporting “Minimally improved,” 3 patients reporting “No change,” and 1 patient reporting “Much worse.”

**FIGURE 4:**
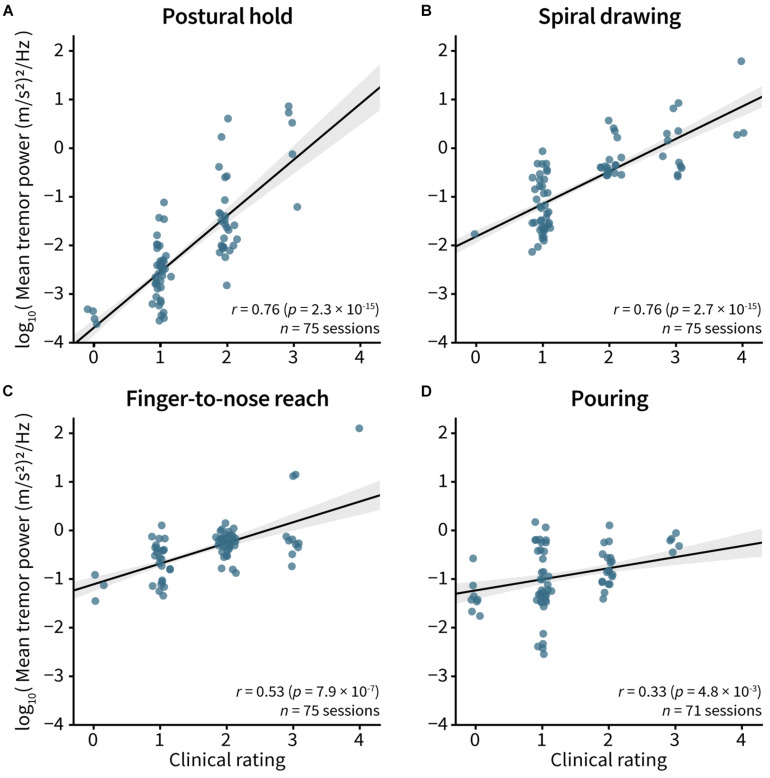
Correlation between tremor power and clinical visual tremor rating. Spearman’s correlation between the FTM-CRS visual rating and the log_10_ tremor power was computed for the **(A)** postural hold, **(B)** spiral drawing, **(C)** finger-to-nose reach, and **(D)** pouring tasks. Each point represents single measurement, with five measurements per patient. The clinical ratings in the plots have been adjusted very slightly to improve visibility of each point. In all plots, line and gray bands represent the best fit line and 95% confidence interval.

Tremor powers were correlated to clinical visual ratings for the postural hold (*r* = 0.76, *p* = 2.3e–15; Figure 4A), spiral drawing (*r* = 0.76, *p* = 2.7e–15; Figure 4B), finger-to-nose reach (*r* = 0.53, *p* = 7.9e–7; Figure 4C), and pouring (*r* = 0.33, *p* = 0.0048; Figure 4D).

No adverse events were reported.

## Discussion

This study characterized the time-profile of tremor reduction during and following non-invasive stimulation (TAPS) of median and radial nerves. The study showed more than 70% of patients received at least 60 min of therapeutic benefit after end of stimulation. Significant and persistent tremor reductions were consistently reproduced across gold-standard clinical ratings (Figure 2) and objective accelerometer measurements (Figure 3). No adverse events were reported, suggesting a favorable safety profile for TAPS. These findings suggest that TAPS could be a safe and effective therapy option for treating hand tremor symptoms in some ET patients.

The post-stimulation duration of benefit objectively measured in this study is comparable to patients’ self-reported durations of benefit in other studies. In a recent 3-month clinical trial that tracked 205 patients using TAPS therapy twice daily at home, 64% of patients reported their tremor relief persisted beyond the end of stimulation (Isaacson et al., 2020). For these patients reporting extended relief, the median self-reported post-stimulation duration of benefit was 60 min (Isaacson et al., 2020).

Similarly, the magnitude of acute (i.e., immediately post-stimulation) tremor improvement reported in this study is consistent with the acute tremor improvements observed in previous TAPS studies. This study found an average FTM-CRS improvement of 0.6 points immediately after stimulation (Figure 2), which was comparable to improvements reported in other studies using similar clinical rating scales [0.3–0.8 point improvement in various upper limb tasks in the Tremor Research Group Essential Tremor Rating Assessment Scale (Elble, 2016), and 0.3–1 point improvement in the Bain & Findley Activities of Daily Living scale (Bain et al., 1993; Pahwa et al., 2019; Isaacson et al., 2020)]. The median 3.2-fold improvement (70% reduction) in postural hold tremor power immediately after the end of stimulation (Figure 3A) in this study is comparable to the range of postural hold tremor power improvements reported in a 3 month at-home clinical trial (Isaacson et al., 2020).

These tremor reductions with TAPS are comparable to the 50–70% tremor reductions reported with first-line ET medications (Hedera et al., 2013). Medications have longer duration of tremor relief (Gironell et al., 1999) than the 60 min duration of benefit measured in the current study, but only approximately half of patients respond to medication and many patients reduce or discontinue use of medications due to the side effects present at the doses required to sufficiently treat tremor (Koller and Vetere-Overfield, 1989; O’Suilleabhain and Dewey, 2002; Zesiewicz et al., 2005; Pal, 2011; Hedera et al., 2013). While TAPS has been shown here and in other studies to have a much lower adverse event rate than medications (Lin et al., 2018; Pahwa et al., 2019; Isaacson et al., 2020), this single-session study alone is insufficient to conclude whether 60 min of benefit after 40 min of stimulation is sufficient for some patients to adopt TAPS therapy into their tremor treatment routine. Future work to examine patient satisfaction with receiving 60 min of benefit after each therapy session, the time course of repeated TAPS throughout the day (i.e., mimicking medication usage), and how TAPS therapy interacts with medication usage would provide valuable guidance for physicians prescribing only TAPS, only medications, or TAPS and medication combined. Similarly, future studies characterizing the trade-off between TAPS therapy duration, stimulation intensity, and post-stimulation duration of benefit may further improve therapeutic outcomes. Mechanistic studies to identify how TAPS modulates neural circuitry to provide extended duration of benefit would be valuable for the field and may provide insights applicable for improving DBS.

This study quantified tremor reduction using both visual FTM-CRS scores and accelerometer-measured tremor power. The former metric is a gold standard assessment for the field, but requires an expert rater, has limited inter- and intra-rater reliability (Stacy et al., 2007), and could be difficult to administer at home. There has been increasing interest in using mobile sensing technology to monitor tremor status, as sensor-based metrics may reduce subjectivity and variance in evaluating tremor severity and provide opportunities for remote monitoring of tremor status (e.g., Pulliam et al., 2014; Daneault, 2018). This study’s outcome metric, tremor power, has been used previously as a measure of tremor severity (e.g., Elble et al., 2006; Daneault et al., 2012; Zheng et al., 2017). This study validated that the measured tremor powers were correlated with gold-standard clinical severity ratings across multiple tasks (Figure 4) with correlation strengths similar to previous reports (e.g., Daneault et al., 2012). Different tasks had different degrees of correlation, possibly due to variability in how clinical ratings and sensor measurements reliably captured tremor characteristics in each task. Our data suggest that certain tasks, such as postural hold, may be better suited to accurately capture information about tremor severity, but future research to develop digital markers that capture tremor severity across a range of tasks would be valuable.

Limitations of this study should be considered while interpreting its results. First, while most patients showed improvement in tremor with TAPS therapy, the degree of improvement was variable between patients and between tasks. This variability is not surprising given the heterogeneity observed in the ET population (Elble, 2017) and the variability in response to current standard-of-care medications and other treatments (Ferreira et al., 2019). Previous work suggests that the hallmark symptoms of ET (kinetic and postural tremors) are driven by multiple central nervous system pathophysiologies and that various tremor tasks may elicit tremor through different sensorimotor pathways (Calzetti et al., 1987; Louis et al., 2006; Schuhmayer et al., 2017). It is possible the variability in these underlying mechanisms affect how each participant and task respond to stimulation. Furthermore, each participant’s stimulation frequency was calibrated to their postural hold tremor frequency. It is possible the increased tremor power reductions observed in the postural hold task are related to this task-specific calibration. Future work to better characterize if and how stimulation therapy should be tuned to ET subtype and task may further improve treatment efficacy.

Second, this was a small, single-session study with safety and efficacy evaluated out to 60 min after a single 40 min TAPS therapy session. The observed tremor reduction was still present at 60 min after end of stimulation for most patients, but longer periods of monitoring or variable duration and amplitude of stimulation are needed to fully characterize the duration of effect. It is also possible that multiple consecutive stimulation sessions (i.e., within and across days) have an interactive effect on tremor reduction and may alter the duration of effect that was observed with a single isolated stimulation session. Future work to develop passive tremor severity monitoring algorithms using wearable motion sensor data (e.g., from a smartwatch) can enable larger-scale studies to objectively track duration of TAPS therapeutic effect at home.

Finally, this study was too small to evaluate the impact of patient characteristics, including age, gender, and medical history, on duration of symptomatic relief following TAPS therapy. All patients in this study remained on their standard-of-care ET treatment. While TAPS has been shown to provide effective symptomatic relief for patients both on and off tremor medication (Isaacson et al., 2020), it is possible the 5 of 15 subjects in this study who were on medication may have ingested medication that could have influenced the measured duration of effect.

This study provides evidence to support that TAPS therapy has a safe and durable effect on ET out to at least 60 min following stimulation, and suggests sensor data recorded before, during, and immediately following stimulation can be used to understand therapeutic response in an at-home setting.

## Data Availability Statement

All datasets generated for this study are included in the article/Supplementary Material.

## Ethics Statement

The studies involving human participants were reviewed and approved by the Western Institutional Review Board. The patients/participants provided their written informed consent to participate in this study.

## Author Contributions

JS-N, KR, ER, and SD conceived and designed the study. JS-N, DK, and ER collected the data. JY, AR, SS, and ER conducted the data and statistical analyses. JY, AR, and ER developed the figures. JY, AR, KR, ER, and SD wrote the manuscript. All authors reviewed and critiqued the manuscript.

## Conflict of Interest

The authors declare that this study received funding from Cala Health, Inc. and that authors are all either employed by are consultants of Cala Health: AR, JS-N, and SS were employed by Cala Health. DK was a consultant for Cala Health. KR was an employee and board member of Cala Health. JY and ER were former employees of Cala Health. SD was a consultant, scientific advisor, and board member of Cala Health. The funder had the following involvement with the study: study conception and design, data collection, data analysis, decision to publish, and preparation of the manuscript (see Author Contributions for details).

## Supplementary Material

Supplementary Figure 1 Per-Patient fold-improvement in tremor power. Tremor power was calculated from accelerometer data at each assessed time for the **(A)** postural hold, **(B)** spiral drawing, **(C)** finger-to-nose reach, and **(D)** pouring tasks. Fold improvement in tremor power is the ratio between tremor power at baseline and the specified time, with a ratio >1, =1, and <1 indicating improvement, no change, and worsening from baseline, respectively. Points and error bars represent log-transformed mean ± 2 standard errors across patients (*n* = 15 for postural hold, spiral drawing, and finger-to-nose reach; *n* = 14 for pouring), and gray lines represent tremor power trajectories per patient.
