# Supplementary figures and images for: Transcutaneous Afferent Patterned Stimulation Therapy Reduces Hand Tremor for One Hour in Essential Tremor Patients
Source: Front Neurosci. 2020 Nov 12;14:530300. doi: 10.3389/fnins.2020.530300 (PMC7689107; doi:10.3389/fnins.2020.530300)

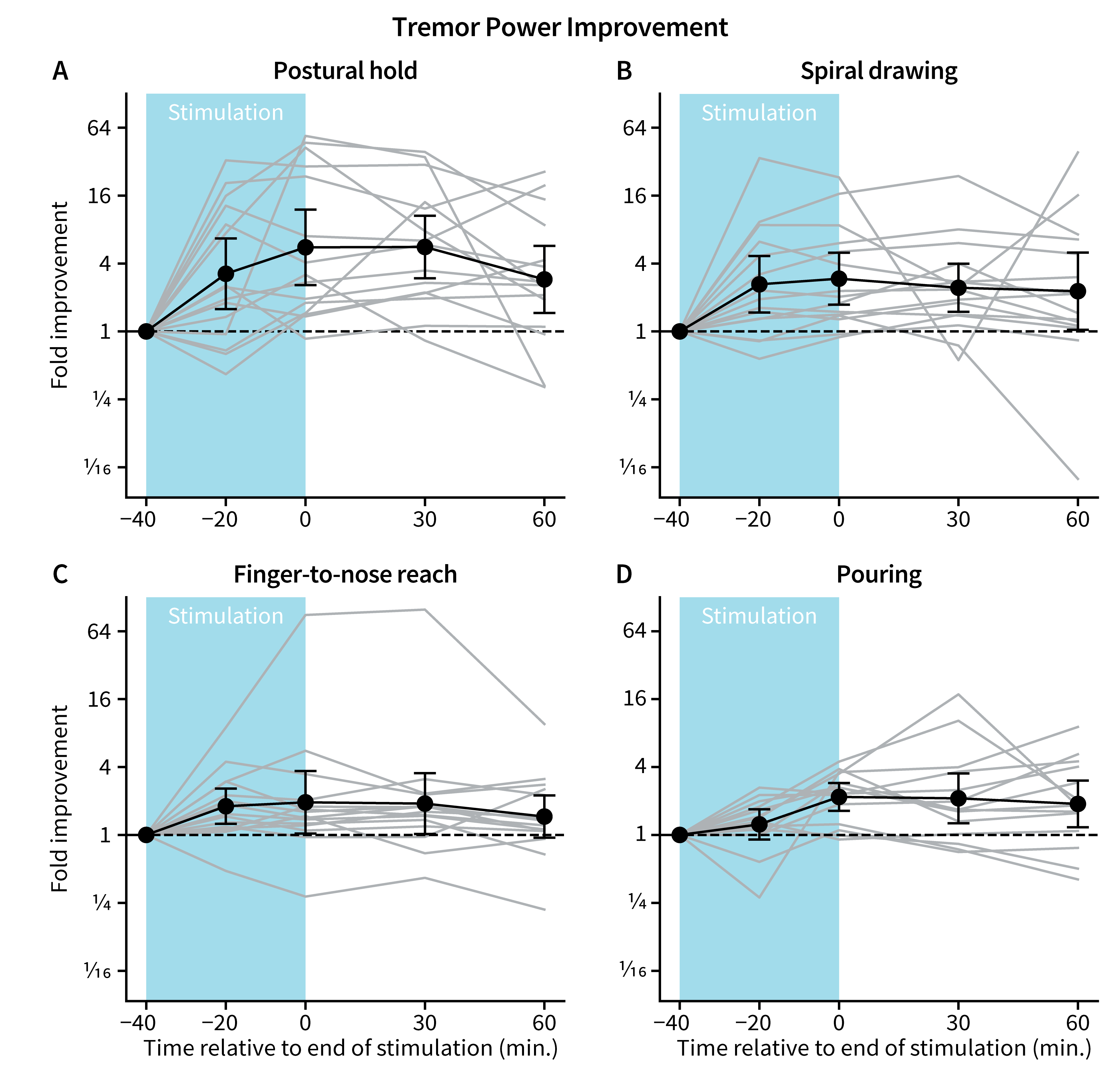

Supplement: Supplementary Figure 1 — Per-Patient fold-improvement in tremor power. Tremor power was calculated from accelerometer data at each assessed time for the (A) postural hold, (B) spiral drawing, (C) finger-to-nose reach, and (D) pouring tasks. Fold improvement in tremor power is the ratio between tremor power at baseline and the specified time, with a ratio >1, =1, and <1 indicating improvement, no change, and worsening from baseline, respectively. Points and error bars represent log-transformed mean ± 2 standard errors across patients (n = 15 for postural hold, spiral drawing, and finger-to-nose reach; n = 14 for pouring), and gray lines represent tremor power trajectories per patient. [file Image_1.tif]
